# The influence of ions and humidity on charging of solid hydrophobic surfaces in slide electrification†

**DOI:** 10.1039/d3sm01153d

**Published:** 2023-12-15

**Authors:** Suhad Sbeih, Aziz Lüleci, Stefan Weber, Werner Steffen

**Affiliations:** a School of Basic Sciences and Humanities, German Jordanian University Amman 11180 Jordan; b Max Planck Institute for Polymer Research Ackermannweg 10 55128 Mainz Germany steffen@mpip-mainz.mpg.de

## Abstract

Water drops sliding down inclined hydrophobic, insulating surfaces spontaneously deposit electric charges. However, it is not yet clear how the charges are deposited. The influence of added non-hydrolysable salt, acid, or base in the sliding water drops as well as the surrounding humidity on surface electrification and charge formation is also not yet fully understood. Here, we measure the charging on hydrophobic solid surfaces (coated with PFOTS or PDMS) by sliding drops with varying concentration for different types of solutions. Solutions of NaCl, CaCl_2_, KNO_3_, HCl, and NaOH, were studied whose concentrations varied in a range of 0.01 to 100 mM. The charge increased slightly at low concentrations and decreased at higher concentrations. We attribute this decrease to the combined effect of charge screening as the non-hydrolysable salt concentration increases and pH driven charge regulation. The effect of humidity on the measured charge was tested over the range from 10% to 90% of humidity. It was found that the influence of humidity on the charge measurements below 70% humidity is low.

## Abstract 2

Water drops sliding down inclined hydrophobic, insulating surfaces spontaneously deposit electric charges.

## Graphical abstract

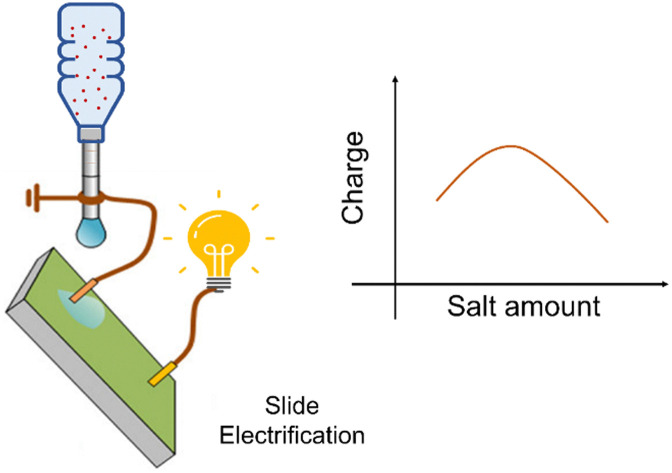

## 1 Introduction

Generating electricity out of thin air might be a dream but some early papers reported the generation of charges by splashing water,^1,2^ waterfalls or even rain.^3^ In recent years generation of charges by sliding drops^4–6^ over specific surfaces or by droplets impacting or being squeezed,^7,8^ has been realized. The electrical charge generated by water drops contacting a solid surface can be used to generate electric currents. As one application a self-powered water droplet sensor based on a flow-through front surface electrode has been demonstrated.^9^ Recent studies of the contact or slide electrification at the liquid–solid interfaces have investigated the questions the role of the electric double layer (EDL, Debye layer) and what and how the charge transfer between the droplet and the surface occurs.^10,11^ A transition from positive to negative charge of sliding water drops on insulating hydrophobic solid surfaces coated with amine-terminated silanes was observed.^6^ The change of the charge polarity on the drops depends on the time between successive drops. This was postulated to be due to the remaining protons accepted by the amine functionalized groups on the surface. Pre-charged hydrophobic surfaces have been used by a homogenous electrowetting-assisted charge injection method and a charge-trapping based electricity nanogenerator for energy harvesting from water droplets was proposed.^12^

In electrokinetic experiments^10^ comparing the influence of salts and dissolved CO_2_ on a range of chemically different surfaces the influence of CO_2_ clearly dominated. They investigated the effect of salt concentration on the surface charge and found that the zeta potential and the surface charge of the surface decreased when a non-hydrolysable salt was added. To explain the role of ionic strength or pH on surface charges and diffuse or EDL potential some researchers refer to the terminology of ‘charge regulation’ or ‘charge induced regulation’.^13–16^ The aspect of different concentrations of solutions modifying the surface charge on the charge regulation has been recently treated theoretically.^16^

The influence of NaCl concentration on charge transfer when a water-front moves across a junction between a hydrophobic dielectric and a metal electrode has been studied.^17^ They performed experiments in combination with simulations that showed that the electrode voltage changes due to the dipping process.

By dipping a metal electrode coated with the hydrophobic polymer fluorinated ethylene propylene into water solutions of different ion concentrations, the charge transfer as a function of ion concentration was measured.^18^ They concluded that the ion specificity does not play a role, with exception to hydrogen ions. They explained their results based on the ion transfer from the electrical double layer model.

Sosa *et al.*^19^ studied the liquid-polymer contact electrification of sliding water drops on a surface as a function of pH and ionic strength of the drops. They found that the addition of non-hydrolysable salts (NaCl, CaCl_2_) decreases the charge induced in the drop. They suggested proton or hydroxyl transfer from the liquid to the hydrophobic polymer surface and proposed a thermodynamic model explaining the transfer process.

Sosa *et al.*^20^ proposed a mathematical model for the liquid-polymer contact electrification of sliding water drops on a hydrophobic surface, that takes into account the charge density and the concentration of added salts. The same model is used to fit the zeta-potential as a function of pH which considers the acid–base equilibrium at the water-polymer interface. For constant pH, they described the decrease of charge and zeta-potential with the concentration of ions by a quenching of water activity at the polymer-water interface.

Despite recent efforts at both experimentally as well as theoretically some basic questions behind the slide electrification are still not completely solved. What is the influence of added salt in the liquid or pH on the generation of charges and the electrification and if and how much does the humidity in the surrounding gas plays a role.

Here we show the influence of concentration of different ionic solutions on slide electrification on the hydrophobic surfaces that have been widely used by us and others in the past. In the past there have been humidity studies on surfaces with polymers^19^ and on contact electrification of ionic electrets.^21^

Apart from the salts studied so far, we studied solutions ranging in pH from a strong acid, HCl (pH = 1), to a strong base, NaOH (pH = 13), and three solutions of non-hydrolysable salts, NaCl, CaCl_2_, and KNO_3_. The concentration of the five solution types was varied from 0.01 to 100 mM (mol L^−1^). The experiments were carried out under control of the humidity; for this purpose, an experimental setup able to work under fixed humidity was created.

## 2 Experimental section

### 2.1 Substrates and surface treatment

Microscope slides of soda-lime glass (26 mm × 76 mm × 1 mm, Thermo Fisher Scientific Gerhard Menzel B.V. & Co. KG, Germany) were hydrophobized by chemical vapor deposition with trichloro(1*H*,1*H*,2*H*,2*H*-perfluorooctyl)silane (PFOTS, Thermo Fisher Scientific)^22^ or by coating with poly-dimethylsiloxane (PDMS) brushes by ‘grafting from’ using dimethyldichlorosilane (Sigma-Aldrich) as monomers.^23^ The reaction time for the polymerization was 1800 s. According to previous work^23^ a thickness of 7 ± 1 nm of the PDMS brush layer is to be expected. In the polymerisation reaction no catalyser or other initiator was added, it is a polyaddition reaction. Therefor no additional ionic components were added in the process which could influence the experiment.

Before being coated, the glass slides were first cleaned with acetone and ethanol in an ultrasonic bath, then treated with an oxygen plasma (Diener electronic FEMTO, Plasma-Surface-Technology, Germany, 6 cm^3^ min^−1^ oxygen flow rate, 120 W) for 600s to remove organic contaminations and to activate the surface. Using stock solutions of concentration 1 M (mol L^−1^) for NaOH, NaCl, CaCl_2_ (VWR, Germany), KNO_3_ (ROTH, Germany), and HCl (J T Baker Avantar, Germany) and deionized water (DI water, 18 MΩ cm, obtained from an Arium® Pro (Satorius, Germany)), solutions of concentrations of 0, 0.01, 0.1, 1, 10, 100 mM were prepared.

### 2.2 Charge experiments

A custom made setup^5^ inside of a grounded Faraday cage was used. The hydrophobic substrates were attached to an electrically grounded copper plate with an inclination of 50 degrees. A peristaltic pump (Gilson, MINIPULS 3, Wisconsin, USA) was used to control the dripping rate with one drop every 2.5 s. The tubing (PVC, Gilson, USA) was connected to a custom-made electrically grounded, blunt needle with an inner diameter of 2 mm to create drops of 45 μL that fell onto the substrate from a height of 5 mm. These drops slid approximately 10 mm before touching a ground wire to neutralize any charges in the drop and a further 40 mm before hitting a probe wire to measure the acquired charge. The probe wire (gold coated Tungsten) was connected to a current amplifier (DDPCA-300, FEMTO, Germany, electrical resistance of 10^6^ Ω).

Before each experiment, the surface was neutralised for 5 minutes with an ionizing air blower (IAB, Mini Zero Volt Ionizer 2, USA, not shown in the schematic picture). The tubing used to transfer the pumped solution to the needle, was flushed with the solution to be used before each experiment. Humidity and temperature were monitored throughout the experiments. The humidity was between 50–70% with the ionic solutions.

**Fig. 1:**
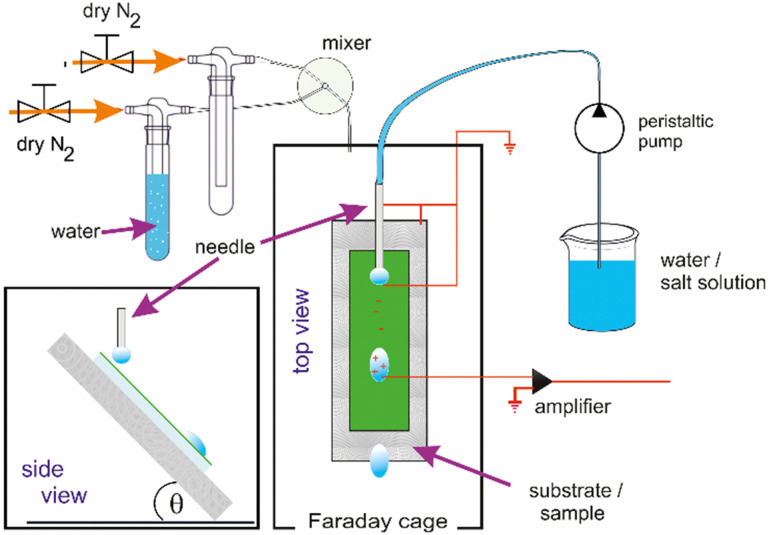
Schematic (top and side view) of the setup to measure drop charges with humidity control.

A second setup^24^ was constructed In order to investigate a possible effect of humidity. The principal layout of the setup described above was kept and humidity control added. To control the humidity in the now airtight experimental chamber, a stream of dry nitrogen was mixed with a stream of nitrogen, that passed a wash-bottle filled with DI water to be saturated with water vapor. The flows were regulated by gas mass flow controllers (FMA5520A, Omega, USA) and a custom-built PID humidity controller before entering the sealed box in the Faraday cage (Fig. 1).

The humidity is controlled in the range from 10% to 90% with an accuracy of 3%. The evaporation from the droplets does not permit reliable dryer conditions in the relatively small volume of our experimental chamber. The temperature of the gas inside of the experimental chamber was monitored by a PT100 and the humidity with a sensor (SH15, Sensirion AG, Switzerland). Both sensors were connected to the custom-built PID controller.

**Fig. 2:**
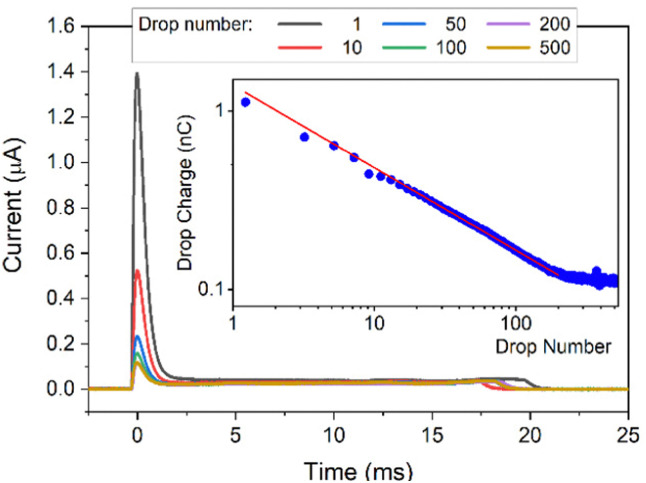
DI water drops containing 10 mM NaOH sliding down a PFOTS substrate. Main figure: Current *versus* time. Insert: drop charge *versus* drop number, the red line is a fit to a power law.

In each experiment, 500 drops were run successively over the surface and a current spike was recorded (Fig. 2) as each drop touched the probe. The drop charge was determined by integrating the first 25 ms of the current. In this way the accumulated charge in the drop is detected. As previously observed, the first drop carried the highest charge. It then decreased and reached a saturation after about 200 drops (when the charge reaches a constant value, Fig. 2, insert, see Fig. S1 (ESI†) for a lin–lin representation of the drop charge). The drop charge depends on how many drops were previously deposited and run down the slope at a given drop interval. This indicates that here is a feedback mechanism leads to self-inhibition. This can be described by a power law *y* = bl + a*x^b^ with a slope *b* = −0.45 and a baseline bl = 0.114 nC. Remaining Charges which have not yet been annihilated through, *e.g.* by ions from the air, hinder the further deposition. ‘Saturation’ is reached when a balance between charges annihilated on or moved away from the surface (in the interval between drops) and creating new charges on the surface is established.

### 2.3 Contact angles and velocity of drops

For characterisation, advancing and receding contact angles of sessile drops were measured with a contact angle goniometer (OCA35, DataPhysics Instrument GmbH, Germany) before the start of the charge measurements, during (right after performing charge measurement with the highest concentration), and after all the charge measurements with all concentrations of the corresponding solution type.

A setup,^25,26^ equipped with a high-speed camera was utilized to capture high resolution videos for the sliding drops of different types of solutions and concentration on PFOTS samples. The average drop velocity and contact angle were determined to study the effect of solution type/concentration on the macroscopic sliding velocity of the droplets and advancing contact angle.

## 3 Results and discussion

### 3.1 Charge *vs.* pH for different solutions

**Fig. 3:**
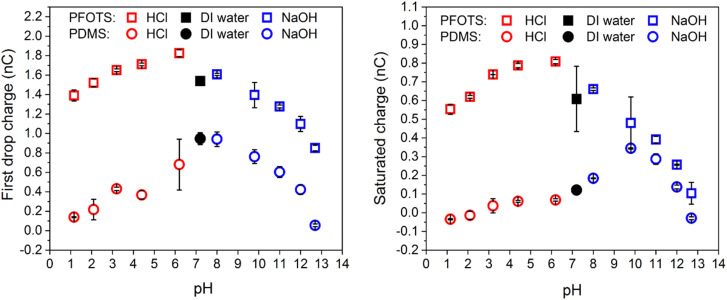
(a) Charge of the first drop *vs.* pH and (b) after the charge reaching saturation *vs.* pH for the cases NaOH as strong base and HCl as strong acid.

When changing the pH value, measured with a pH meter (ESI,† Analytics Lab855, probe: Hamilton, Fig. S2), by adding HCl or NaOH, we observed a maximal charge generation around neutral pH and decreasing charging at low and high pH (Fig. 3) both, for the first drop and the saturated charge. The only exception is the saturated charge on PDMS. We hypothesise that the shift of the maximal charging to pH 10 is caused by the mobility of the hydroxyl ions deposited on the PDMS. As a result, the surface is neutralized faster after a drop has passed. For the surface with PDMS, the trends are not as clear.

However, at high HCl or NaOH concentrations the pH charge regulation at the surface dominates the net charge on the sliding drop. The peak at low HCl or NaOH concentrations can be attributed to the combined effect of both charge screening due to the existing salts in water and the pH charge regulation at the surface.

### 3.2 Charge *vs.* concentration for different solutions

**Fig. 4:**
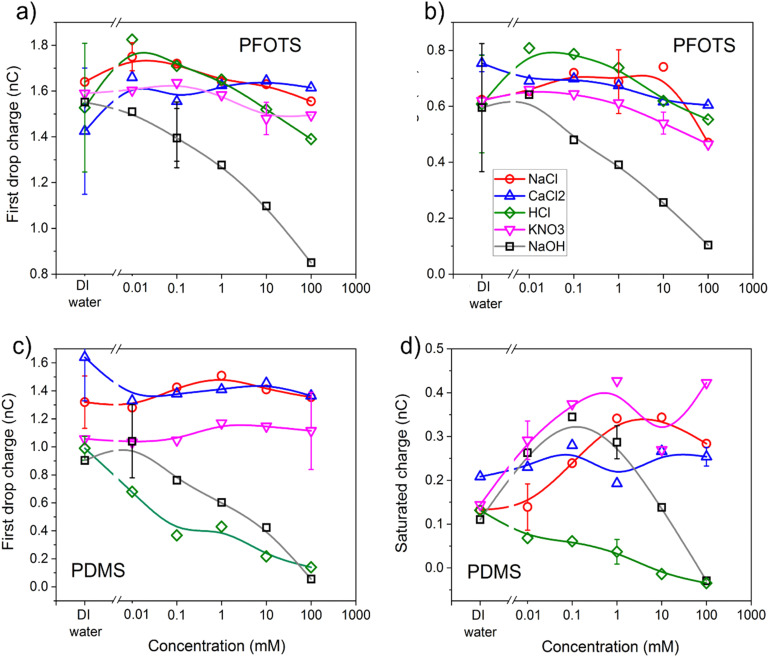
Charge of the first drop and the saturated charge (average of drops 200–500) *vs.* the concentration of the five solutions. (a) Charge of the first drop on PFOTS-coated glass. (b) Saturated charge on PFOTS-coated glass (c) Charge of the first drop on PDMS-coated glass. (d) Saturated charge on PDMS coated glass. Lines are guide for the eyes connecting the data with B-splines.

The charge of the first drop and the saturation charge for the three non-hydrolysable salts solutions as well as the strong acid and base solutions on PFOTS and PDMS samples as a function of the concentration of the solutions are plotted in Fig. 4. As has been observed before,^5,27^, the charge of the first drop was higher than the saturated charge on both PFOTS and PDMS substrates. The saturated charge on PFOTS was higher than that for PDMS coated substrates.

On PFOTS coated substrates, both the charge of the first drop and the saturated charge first increases slightly as the concentration of the three non-hydrolysable salts increases, showing a small peak around 0.1 mM followed by a weak decrease towards higher non-hydrolysable salt concentration. The only exception is NaOH, which showed a substantial decrease at higher base concentration and thus high pH. For the PDMS coated substrates, the charge of the first drop does not depend significantly on the concentration of the three non-hydrolysable salts (NaCl, CaCl_2_, and KNO_3_). The substantial decrease of the charge of the first drop with the NaOH and HCl concentrations means that the charge is higher at neutral pH. For the saturated charge the trend is not as clear, which may be due to a different mobility of deposited ions on the flexible PDMS layers.

In the recent past have several attempts have been made to model the charge separation and the charging of the surface based on surface chemistry.^19,20,28^ Another explanation which at least partially could play a role is based on electron transfer and charge separation models have been proposed.^4,29^

Looking at the surface chemistry, in water due to the autoprotolysis^30^ hydroxide ions are produced as well as their positively charged counterparts. This is the prerequisite to form the EDL of bound surface charges and their counter charges when the water is brought into contact with an uncharged surface. The thickness of this EDL, which is described by the Debye length *λ*, is between 1 and 1000 nm depending on salt concentration. The EDL itself is macroscopically neutral.

1 H_2_O ↔ HO^−^ + H_3_O^+^

These hydroxide ions can react with the surface, or the dissociation takes place as part of the reaction *e.g.* for glass and quartz with their SiO_2_ networks:^20,31^2
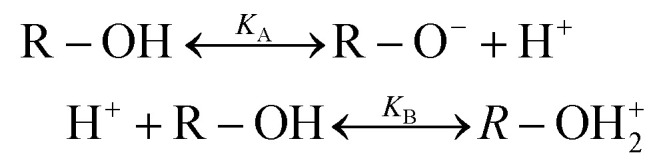


For surfaces of polymers or proteins with groups that can dissociate at least part of the EDL can be formed from these ions. How does the EDL form for our two systems lacking this dissociation property? An explanation is given by Mayrhofer *et al.*^32^ for the fully fluorinated surface PFOTS and by Kudin *et al.*^33^ for the fully hydrogenated PDMS surfaces. Both are hydrophobic whereby PFOTS obtains its hydrophobicity through what is termed “polar hydrophobicity”.^32,34^ A positive affinity for OH^−^ ions for hydrophobic surfaces has been found.^33^ The difference in polarity of these end groups end hence their possible different affinity for OH^−^ could be an explanation for the difference in charging behaviour. Condensation of OH^−^ to the surfaces to form the EDL would at least explain why the surfaces are negatively charged after charge separation and deposition and the droplets are positively charged for our two systems (the chemical structures of the two molecules can be found in Fig. S3, ESI†). A complete study how and why the EDL is formed for our systems is beyond the scope of this paper and subject of further studies.

In general, we have to conclude that the magnitude of charging depends on the surface used. Our results on PFOTS and PDMS differ in some parts by a factor of two and are different from the result of others^6,18–20^ using other surfaces and substrates. We assume, that due to the chemical difference between the substrates a different affinity of OH^−^ ions leads to a difference in the EDL.

An explanation for the separation mechanism at the rear contact line, *i.e.* why the bound surface charges keep separated as the drop moves on, was given recently^28^ in terms of flows at the receding contact line described by the Péclet number (Pe). There is an upward convective flow component found in simulations in the droplet that moves the ions in the diffuse bilayer away from the solid–liquid interface. As a result, the effective screening length is increased by the flow. As long as the convectional flow is stronger than the diffusion of the ions, this leads to an enhancement of the charges on the surface and thus in the droplet as well. The Pe relates convective transport to diffusion. It is given by Pe = *Uλ*/*D*, where *U* is the drop velocity and thus the flow velocity of the liquid at the rear contact line, and *D* is the diffusion coefficient of the ions in the diffuse double layer. Our experimental findings are commensurate with this.

According to Debye–Hückel theory the anion and cation concentrations at a given distance (*x*) from the surface are related to the surface potential (*ψ*_0_) by the Boltzmann factor, *k*_B_, and temperature, *T*, and are given by the equation:^35^3
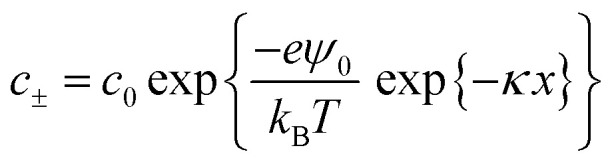
where *c*_0_ is the bulk concentration of ions in particles per m^3^, *e* is the electron charge, and *κ* is the inverse Debye length. The inverse Debye length depends on the bulk concentration of ions and is given by:4
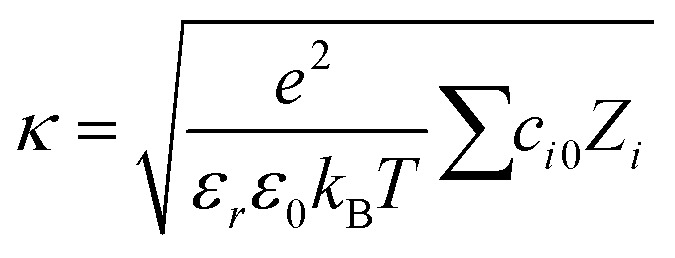


Here, *ε*_r_ is the relative permittivity, *ε*_0_ is the permittivity of free space, (*Z*_i_) is the valency of the *i*th ion type. The total ion concentration at a given distance *x* is given by the difference between the negative and positive ions:5
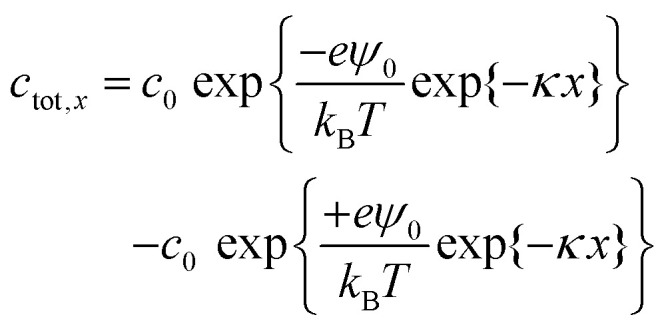


The total charge in the electric double layer can be calculated by integrating the total ion concentration at a small distance (slice) from the surface. The total charge always increases with bulk concentration due to the dominant linear dependence caused by the *c*_0_ multiplied in front of the exponentials. Calculations employing the surface potential function and Debye length were done for fitting the saturated charge behaviour with ion concentration. We find, as the concentration increases, Debye length decreases thus the net charge of the drop increases (Fig. S5, ESI†).

The model in the last paragraph explains the initial increase when *e.g.* HCl is added. The implications of this model are in competition with the increasing screening, so that at higher concentrations the charging decreases.

Another point is the influence of different concentrations of salts, bases, or acids on the amount of charging. Assuming charge regulation^16,36^ at the interface, an increased screening length leads to a reduced surface charge density. As the concentration of non-hydrolysable salt increases, the Debye length in the drop decreases and the charge screening increases leading to a net charge decrease.

**Fig. 5:**
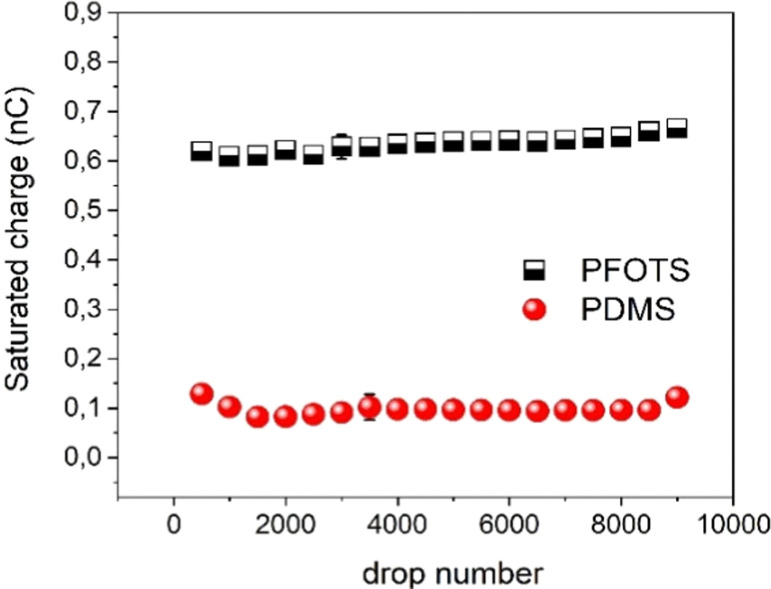
Drop charge after reaching saturation for up to 9000 of DI water drops on PFOTS and PDMS to demonstrate the stability of the surface.

To test the durability of our coated substrates experiments were conducted with 9000 water drops sliding over the coated surfaces to see whether the charge is affected by the large number of sliding drops. The results (Fig. 5) show that the saturated charge remained around the same values for both substrates within the error even after this huge number of sliding DI water drops. From this we conclude that the surface is at least not substantially damaged or altered by the passing water drops.

### 3.3 Humidity effect on charge measurements

**Fig. 6:**
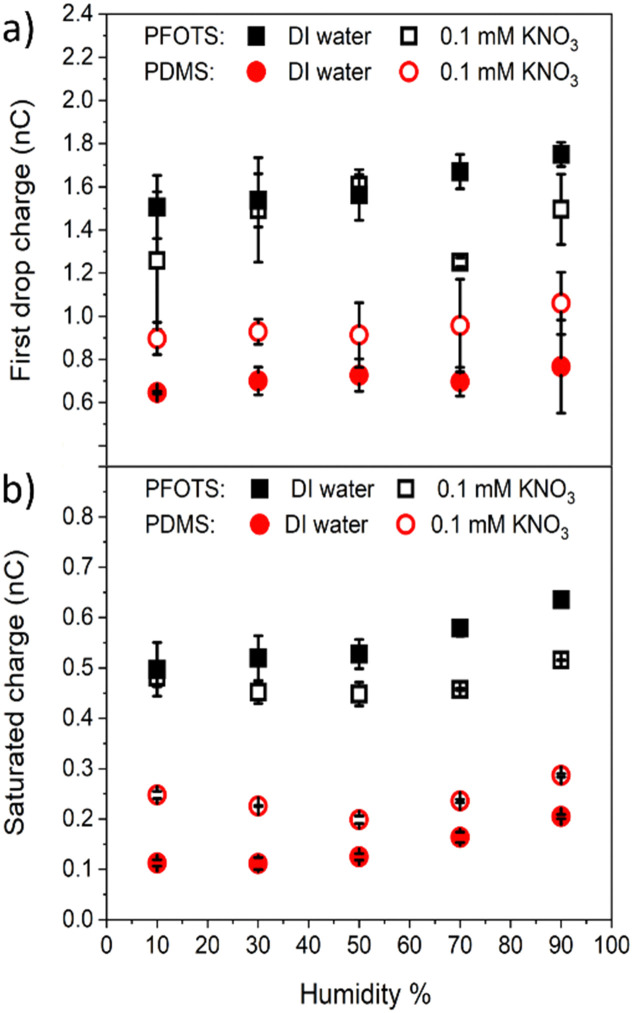
The (a) first drop charge and (b) saturated charge *versus* humidity on PFOTS and PDMS substrates for DI water and 0.1 mM KNO_3_ solution.

The charge on the coated surfaces was measured by employing a setup^24^ built for controlling the humidity inside the chamber in which the samples were mounted. PFOTS and PDMS substrates were tested with DI water and 0.1 mM KNO_3_ solution, respectively. Prior to every experiment the humidity was allowed to saturate for 5 minutes after which the variation was in the range of ±3%. In the range of 10–90% relative humidity we did not observe a substantial dependence of the measured drop charge *versus* humidity (Fig. 6). Our explanation for the lack of humidity dependence is, that in the vicinity of the drop, the air is saturated with water vapor. Since the water vapor molecules in air have a diffusion coefficient of *D* = 2.5 × 10^−5^ m s^−1^ we estimate the length scale over which water vapor equilibrate to be 
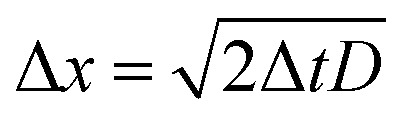
 at a given time Δ*t*. At the same time the drop moves Δ*x* = *U*Δ*t*.

Equating both equations we see that even at a speed of *U* = 0.3 m s^−1^ the region saturated with water vapor is around 200 μm, well ahead or behind the drop.

### 3.4 Effect of exposure to non-hydrolysable salts, strong acidic, and strong basic solutions on water contact angle of hydrophobic substrates

**Fig. 7:**
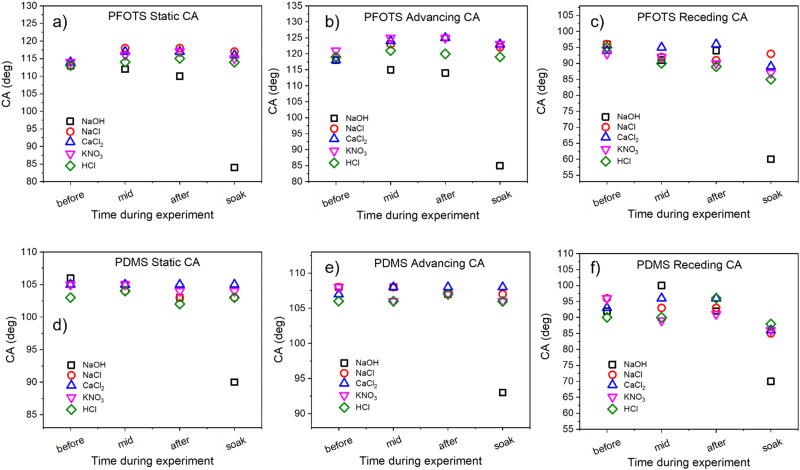
Contact angles (CA) of DI water: (a) static (b) advancing and (c) receding CA of PFOTS on glass and (d) static (e) advancing and (f) receding CA of PDMS on glass before, during, and after charge measurement experiments with different solutions. To look for possible damage the samples were soaked in the respective solutions and CAs measured afterwards.

We investigated the effect of the sliding drops of different solutions on the macroscopic contact angles. That is, to see whether or not the surfaces are being damaged doing the charge experiments. The static contact angle for water *s* are 113° ± 2° and 104° ± 2° for pristine PFOTS and PDMS coated samples, respectively (Fig. 7). The receding and advancing contact angles are in the range of 90° to 120° for the PFOTS and 90° to 110° for PDMS. The static, advancing and receding contact angles for water were measured for all samples before experiments, after experiments, and after soaking them in the 100 mM solution (for 1 h to see the maximum effect). The contact angles were not greatly affected by the sliding drops of any of the solutions apart for the samples being soaked in the 100 mM NaOH solution, here the contact angles decreased significantly. The receding, and advancing contact angles for PFOTS decreased to 60°, and 85°, respectively (Fig. 7). Similarly, the receding, and advancing contact angles for PDMS decreased to 70° and 93°, respectively. NaOH solution has the biggest effect on the water contact angles for both PFOTS and PDMS coated glass substrates. The results at pH 13 should therefor be treated with caution, albeit they follow the general trend in our charging measurements, as etching of the glass surface cannot be ruled out. Only after this long soaking in pH 13 solution the glass is obviously damaged (Fig. 7). It can therefore be concluded that the surfaces have not been damaged during the time of our experiment.

### 3.5 Average front velocity and contact angle of sliding drops of different solutions dependence on their concentration

To characterize our hydrophobic surfaces, measurements for the average advancing contact angle (CA) and the front velocity of the sliding drops of two non-hydrolysable salts, strong acid, and strong base solutions were conducted. PFOTS samples were used to measure the contact angle and the drop velocity for the four solution types (NaOH, NaCl, CaCl_2_, and HCl) as a function of solution concentration.

**Fig. 8:**
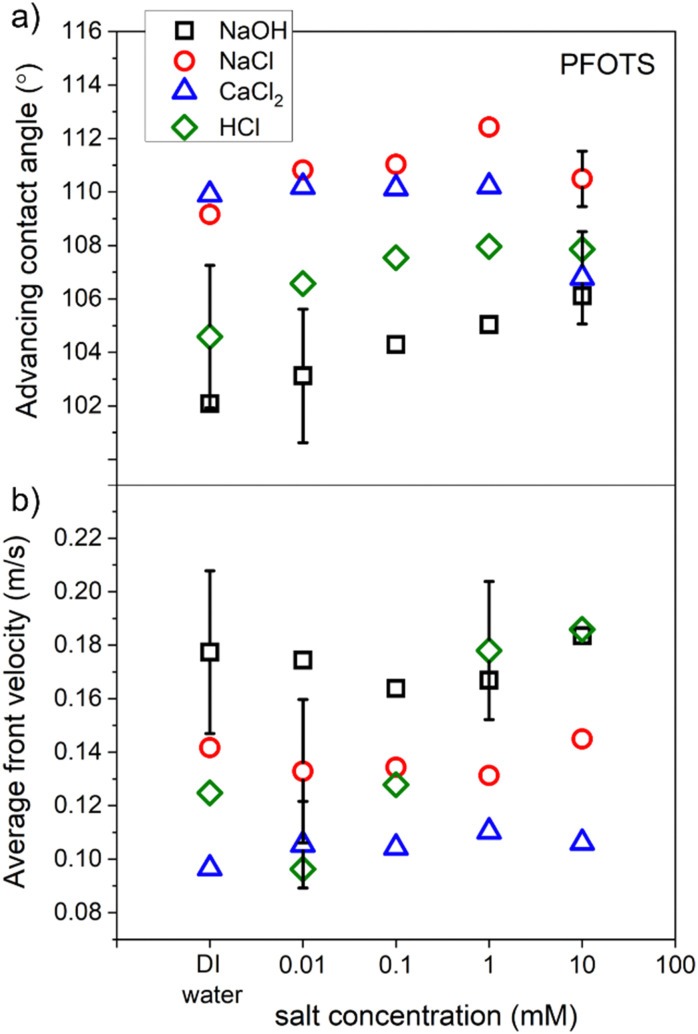
The average of (a) contact angle and (b) front velocity of sliding droplets of four types of solution on PFOTS surfaces as a function of solution concentration.

A video of a sliding drop of each solution on a PFOTS coated substrate that is tilted at 50 degrees, was captured by a high-speed camera. The shape of the droplet, over the captured frames, was fitted with the software supplied with the goniometer and the averages of both the advancing and receding contact angles were obtained. The average front and rear velocities of the sliding drops were also determined from the measured sliding distance. Three sliding drops of each concentration of the four solution types were recorded. Fig. 8 shows the results of the average advancing contact angle and the average velocity of these sliding drops as a function of the concentration of the solution. The results showed that the concentration of a solution did not affect considerably the CA and velocity for all solutions except for HCl. The front velocity of the droplets increased with HCl concentration. NaOH solutions showed the lowest front CA and the highest front velocity. Recently, a slide length dependence of the velocity and the receding contact angle was found for similar substrates and solution's types.^37^

## 4. Conclusions

In this work we have measured the charge generated by sliding drops of five types of solution on hydrophobic solid surfaces. The influence of the different types of solutions and their concentration on the generated charge slide electrification was studied. Glass surfaces coated with PFOTS or PDMS were tested with non-hydrolysable salts, a strong acid, and a strong base in solution at concentrations between 0.01 and 100 mM. Our results show that, compared to sliding drops of DI water, the addition of non-hydrolysable salt, or a strong base at low concentration enhances the charge generation. A charge peak at low concentrations can be attributed to the combined effect of both charge screening as the non-hydrolysable salt concentration increases and pH charge regulation at the surface. Surfaces coated with the PFOTS acquire higher charge than those with PDMS brushes.

The contact angle of the sliding drops of the solutions did not change considerably with increasing concentration of the solutions but was the lowest for NaOH solutions.

The average velocities of the sliding drops didn’t change with increasing concentration of the solutions for most solutions but increased with increasing HCl concentration. The water contact-angles of the tested surfaces did not change considerably during the experiments with the different solutions. It did not change even after being soaked in 100 mM solution for most, but not for NaOH. Only for long time immersion at high pH there was probably damage to the glass inferred.

At a humidity of 10–70% the charge generation is not significantly affected which we explain to be due to the saturation of the air in front and behind the droplet due to the high diffusivity of the water molecules in air.

To summarize, neither variation in pH nor humidity is changing the charging in slide electrification significantly for the two surfaces tested. These are systematic changes which are commensurate with recent results.^28^

## Author contributions

Suhad Sbeih: data curation, formal analysis, investigation, methodology, project administration, visualization, writing – original draft, writing – review & editing; Aziz Lüleci: data curation, formal analysis, methodology; Stefan Weber: methodology, resources, project administration; Werner Steffen: conceptualization, funding acquisition, project administration, resources, software, supervision, validation, visualization, writing – original draft, writing – review & editing.

## Conflicts of interest

There are no conflicts of interest to declare.

## Supplementary Material

SM-020-D3SM01153D-s001
