# Supplementary material for: The influence of ions and humidity on charging of solid hydrophobic surfaces in slide electrification
Source: Soft Matter. 2023 Dec 15;20(3):558–65. doi: 10.1039/d3sm01153d (PMC10792611; doi:10.1039/d3sm01153d)
Supplement: SM-020-D3SM01153D-s001 [file SM-020-D3SM01153D-s001.pdf]

Electronic Supplementary Material (ESI) for Soft Matter.
This journal is © The Royal Society of Chemistry 2023

ARTICLE

The influence of ions and humidity on charging of solid hydrophobic surfaces in slide electrification

Suhad Sbeih[a,b], Aziz Lüleci[b], Stefan Weber[b], and Werner Steffen[*,b]

9. Supplementary information

9.1 Results from experiments

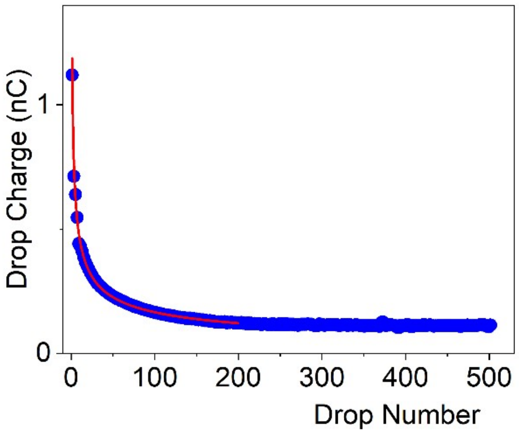

Fig. S 1: drop charge versus drop number from inset of **Error! Reference source not found.** in linear representation. The red line is a power law fit ($y=bl+a*x^b$) with a slope b= -0.45.

The drop charge depends on how many drops have been previously deposited and run down the slope, i.e a feedback mechanism exists that leads to self-inhibition.

9.2 pH measurements

The prepared solutions at different concentrations of the used salts were obtained from stock solutions at 1 M for each salt. A pH meter (SI Analytics Lab855, probe: Hamilton) was used to measure the pH value of each solution. **Fig. S** 2 shows these results. The pH meter was calibrated between each measurement with standard solutions.

9.3 Charge in sliding drop model

The Poisson-Boltzmann equation relates the electric potential and charge density in a solution near a negatively charged plane. The linearized form of the equation (at low potentials) is the one investigated here using Mathematica. The functions of concentrations (CP and CN) of the cations and anions are plotted versus the distance from the surface (x). The arbitrary values (0.1, 0.2, 0.3) are inserted into the bulk salt concentration constant (C0) and the potential value at the surfaces (PSI0). That was done by fixing C0 value and changing PSI0. The area between the two functions over a smal

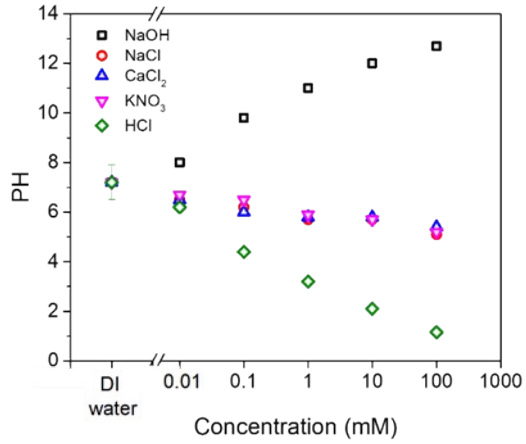

Fig. S 2: pH values of solutions used as a function of concentration.

Tab. S 1: Simulation results of salt concentration and surface potential effect on

PSI	C0	charge
0.1	0.1	0.008
0.1	0.2	0.015
0.1	0.3	0.023
0.2	0.1	0.016
0.2	0.2	0.031
0.2	0.3	0.046
0.3	0.1	0.023
0.3	0.2	0.046
0.3	0.3	0.069

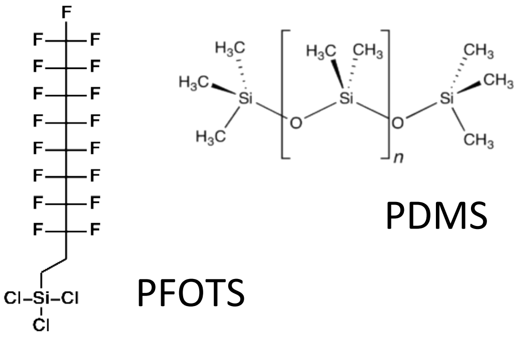

Fig S 3:Chemical structures of PFOTS and PDMS

l distance x (0→1) is calculated by doing the integral of the combined functions. This represents the charge amount acquired by the drop which we are measuring in our experiments. **Error! Reference source not found.**, shows an example when C0 value is fixed at 0.2 and PSI0 is changed over the values (0.1, 0.2, 0.3).

```
In[885]:= Clear[CO, PSIO, CP, CN, Q]
          CO = 0.2;
          PSIO = 0.1;
          CP = CO * Exp[0.4 * PSIO * Exp[-Sqrt[0.03 CO] x]];
          CN = CO * Exp[-0.4 * PSIO * Exp[-Sqrt[0.03 CO] x]];
          Plot[{CP, CN}, {x, 0, 100},
           PlotRange → {{0, 100}, {.15, 0.22}}, AxesOrigin → {0, .15}]
          Print["Area between curves from x=0 to x=1 is"]
          AR = NIntegrate[CP - CN, {x, 0, 1}]
```

Out[890]=
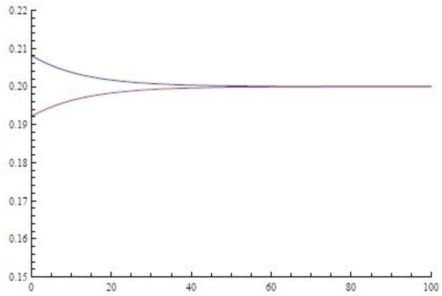

```
Area between curves from x=0 to x=1 is
Out[892]= 0.0153998
```

```
In[893]:= Clear[CO, PSIO, CP, CN, Q]
          CO = 0.2;
          PSIO = 0.2;
          CP = CO * Exp[0.4 * PSIO * Exp[-Sqrt[0.03 CO] x]];
          CN = CO * Exp[-0.4 * PSIO * Exp[-Sqrt[0.03 CO] x]];
          Plot[{CP, CN}, {x, 0, 100},
           PlotRange → {{0, 100}, {.15, 0.22}}, AxesOrigin → {0, .15}]
          Print["Area between curves from x=0 to x=1 is"]
          AR = NIntegrate[CP - CN, {x, 0, 1}]
```

Out[898]=
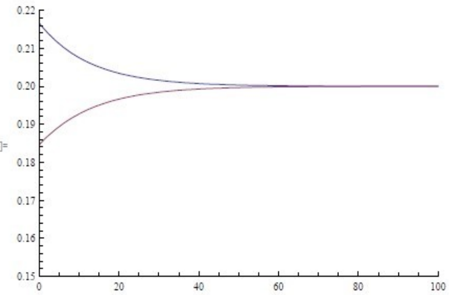

```
Area between curves from x=0 to x=1 is
Out[900]= 0.0308225
```

```
In[909]:= Clear[CO, PSIO, CP, CN, Q]
          CO = 0.2;
          PSIO = 0.3;
          CP = CO * Exp[0.4 * PSIO * Exp[-Sqrt[0.03 CO] x]];
          CN = CO * Exp[-0.4 * PSIO * Exp[-Sqrt[0.03 CO] x]];
          Plot[{CP, CN}, {x, 0, 100},
           PlotRange → {{0, 100}, {.15, 0.23}}, AxesOrigin → {0, .15}]
          Print["Area between curves from x=0 to x=1 is"]
          AR = NIntegrate[CP - CN, {x, 0, 1}]
```

Out[914]=
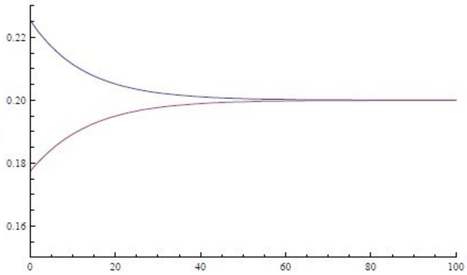

```
Area between curves from x=0 to x=1 is
Out[916]= 0.0462909
```


Fig S 4: Charge calculations using Mathematica.

Increasing the solution's salt concentration (C0 value) leads to an increase in the amount of the charge of a drop. Also, increasing the surface potential (PSI0 value) leads to an increase in the charge. Figures A and B in **Error! Reference source not found.** show these results. All the results are represented in **Tab. S 1**.

The combined effect is shown in the 2D plot, **Error! Reference source not found.**C. The size of the circles indicates the area (the amount of charge) as labelled. The y-axis is the PSI0 values (surface potential) and the x-axis is the C0 values (salt concentration).

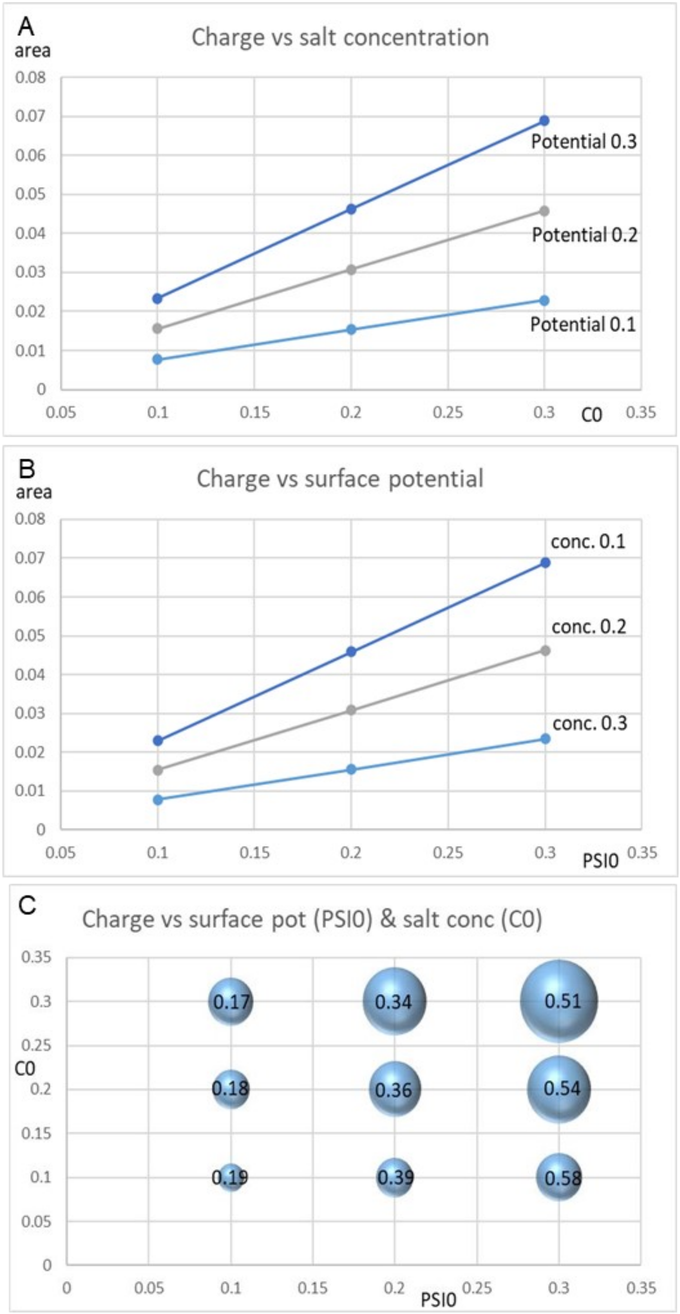

Fig. S5: A- charge versus salt concentration B- charge versus surface potential C- 2D plot of the combined effect on charge.
